# Stable Nuclear Transformation System for the Coccolithophorid Alga *Pleurochrysis carterae*

**DOI:** 10.1038/srep22252

**Published:** 2016-03-07

**Authors:** Hirotoshi Endo, Megumi Yoshida, Toshiki Uji, Naotsune Saga, Koji Inoue, Hiromichi Nagasawa

**Affiliations:** 1 Precursory Research for Embryonic Science and Technology, Japan Science and Technology Agency, Japan; 2 Graduate School of Agricultural and Life Sciences, the University of Tokyo, Japan; 3 Atmosphere and Ocean Research Institute, the University of Tokyo, Japan; 4 Faculty of Fisheries Sciences, Hokkaido University, Japan; 5 Institute for Food Sciences, Hirosaki University, Japan; 6 College of Life Sciences, Zhejiang University, China

## Abstract

Of the three dominant marine microalgal groups, dinoflagellates and diatoms can undergo genetic transformation; however, no transformation method has been established for haptophytes to date. Here, we report the first stable genetic transformation of a coccolithophore, *Pleurochrysis carterae*, by means of polyethylene glycol (PEG)-mediated transfer of a bacterial hygromycin B-resistance gene. Together with the novel transient green fluorescent protein (GFP) expression system, this approach should facilitate further molecular-based research in this phylum.

The contemporary ocean is dominated by the three kinds of microalgae: diatoms (Heterokontophyta), dinoflagellates (Dinophyta), and coccolithophores (Haptophyta). Although successful transformation systems were developed for the former two groups in the late 1990s^1^,^2^, neither a transient nor stable transformation system has been established for Haptophyta until now. Coccolithophores, the main members of this phylum, are algae sharing the common characteristic of intracellular calcification, and cover themselves with fine oval structures of calcite crystals called coccoliths (Fig. 1a). Through coccolith synthesis and photosynthesis, these algae contribute to global carbon circulation^3^,^4^,^5^. A recent pan-genome analysis conducted on a representative coccolithophore species, *Emiliania huxleyi*, revealed extensive intraspecific genome variability, suggesting its potential for investigating the relationship between genomic plasticity and global/historical climate change^6^. In addition to their importance to ocean ecology, there is considerable interest in the commercial application of coccolithophores for bioenergy production. *Pleurochrysis carterae* is regarded as a promising candidate for biodiesel production for the following reasons: a high lipid content up to 33% of their dry weight, capability for long-term outdoor culture, and low potential for contamination of protozoans or other algae owing to the high pH of the growth medium during outdoor cultivation^7^. Given these advantages and the urgent need for alternative sources of biodiesel production, establishment of genetic transformation technologies for haptophytes, especially coccolithophores, is required. Thus, in the present study, we developed the methods of transient and stable transformation for *P. carterae*.

## Results

**Figure 1:**
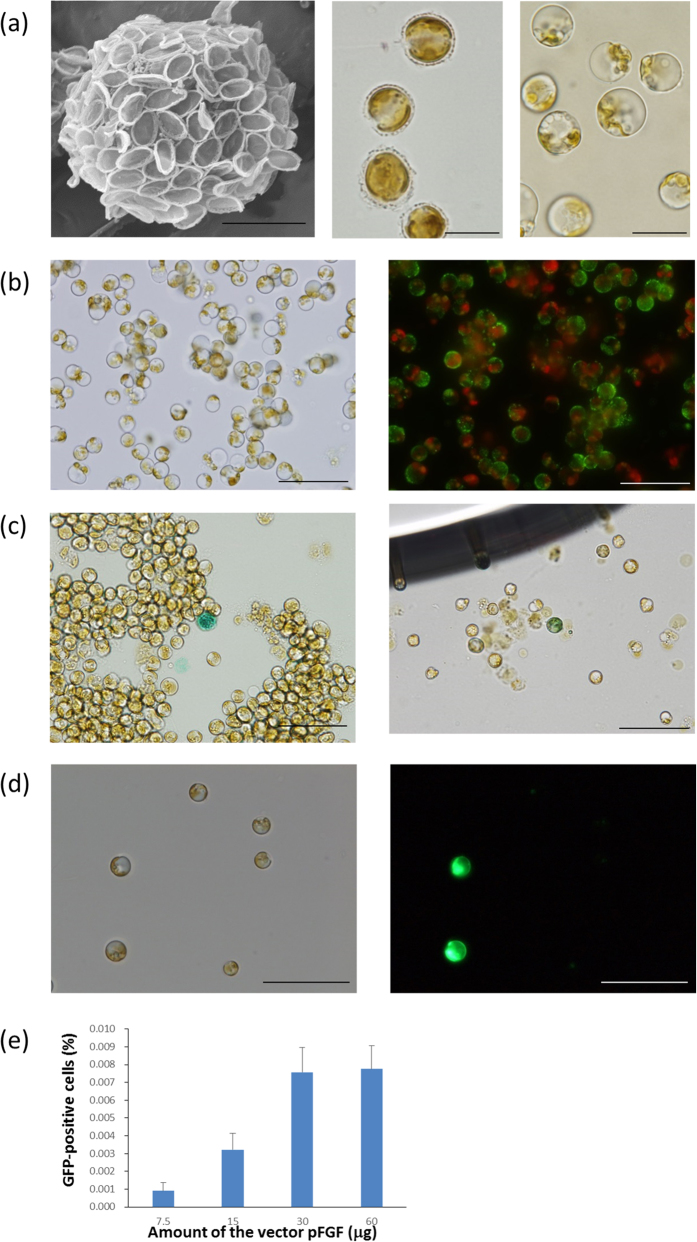
Microscopic views of the coccolithophore, *Pleurochrysis carterae*. **(a)** Left: scanning electron microscopy of a calcified cell. Scale bar, 3 μm. Center: light microscopy of calcified cells; a thick coccosphere can be observed on the cell surface. Right: light microscopy of protoplasts; the coccosphere containing coccoliths is completely removed. Scale bars, 10 μm. **(b)** Fluorescein isothiocyanate (FITC)-labeled dextran-transferred protoplasts. Left: bright-field view. Right: fluorescent view. Green fluorescence can be observed in the cytosol with red fluorescence derived from the chloroplasts. Scale bars, 50 μm. **(c)** Representative transient GUS-expressing cells 3 days after pFGS introduction. Since *P. carterae* cells were sensitive to a detergent (TritonX-100), disrupted cells were frequently observed during the experiment (data not shown). Scale bars, 50 μm. **(d)** Representative transient GFP-expressing cells 24 h after pFGF introduction. Scale bars, 50 μm. **(e)** GFP transient expression analysis. Dose-response relationship between the vector pFGF and GFP-expressing cells. The positive cells were counted at 24 h after vector administration. n = 8, bars represents mean +S.E.

Algal cell walls contain several substances such as elastic or rigid polysaccharides, and mucilaginous, siliceous, cellulose theca, or sulfated polysaccharides^8^. In the case of *P. carterae*, the plasma membrane is coated with a thick layer consisting of as-yet-uncharacterized viscous polysaccharides containing uncalcified coccolith base plates and highly calcified coccoliths. This complicated cell wall structure (generally called the “coccosphere”, corresponding to the cell wall of other algae or higher plants) is the first barrier for effective gene transfer; therefore, removal of the coccosphere and protoplast preparation were the primary objectives for the development of a novel gene transfer system. Takayanagi *et al.*^9^ reported a simple protoplast preparation method for *Pleurochrysis haptonemofera* (a species closely related to *P. carterae*), in which the calcified cells “molt” their coccospheres in a K^+^-containing hypoosmotic buffer. In the present study, we found that pre-treatment with proteinase K was effective for degrading the coccosphere of *P. carterae* cells, and thus a sufficient number of protoplast cells could be easily obtained with our improved method (Fig. 1a). We also found that polyethylene glycol (PEG) treatment could effectively promote the transfer of exogenous macromolecules into the protoplast. Indeed, uptake of fluorescein isothiocyanate (FITC)-dextran was observed in the majority of the PEG-treated cells (Fig. 1b).

**Figure 2:**
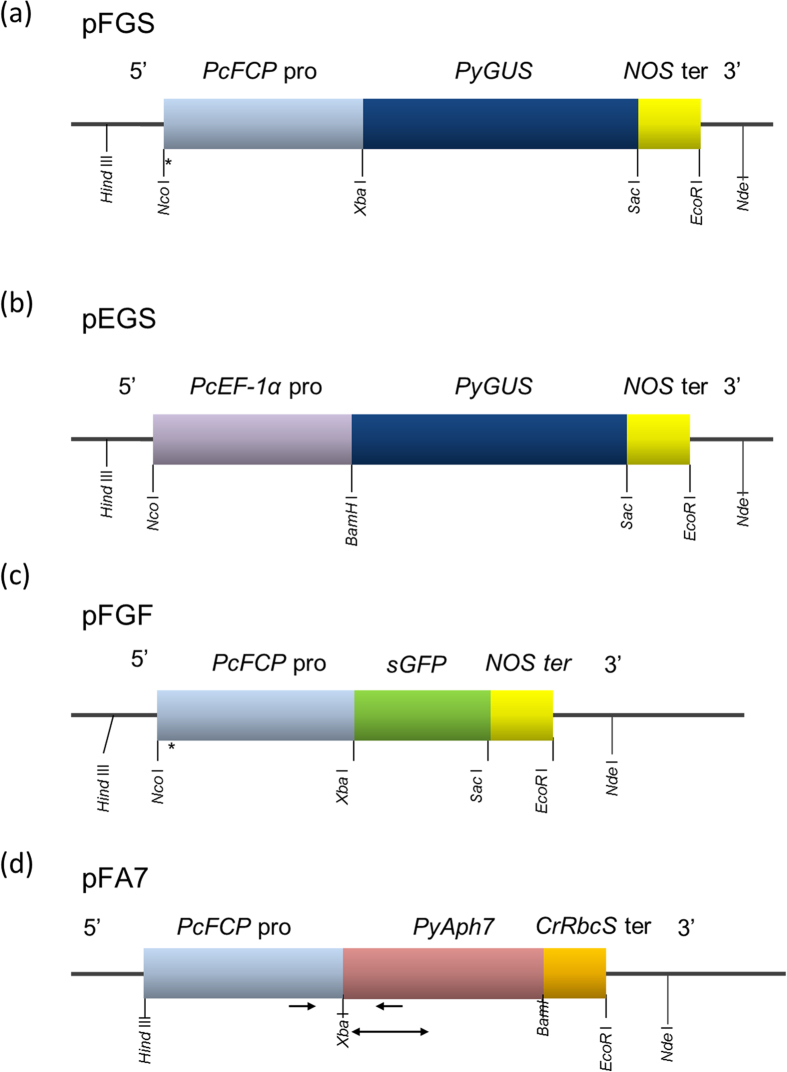
Schematic representations of the expression constructs used in the present study. **(a)** pFGS, **(b)** pEGS, **(c)** pFGF, **(d)** pFA7. *PcFCP*pro, *Pleurochrysis carterae FCP* promoter; *PyGUS*, *Pyropia yezoensis GUS* gene; NOSter, *nopaline synthase* terminator derived from pBI221 vector; *PcEF-1α*pro, *P. carterae EF-1α* promoter; sGFP, a synthetic *GFP* gene; *PyAph7*, a hygromycin B-resistant gene optimized for *P. yezoensis*; CrRbcSter, *Chlamydomonas reinhardtii* ribulose 1,5-bisphosphate carboxylase/oxygenase small subunit terminator. *Twenty base pairs derived from the pGEM-T Easy vector were inserted. Arrows in **(d)** indicate a primer set used in the genomic PCR analysis. The double-headed arrow in **(d)** indicates the probe used in Southern hybridization analysis.

**Figure 3:**
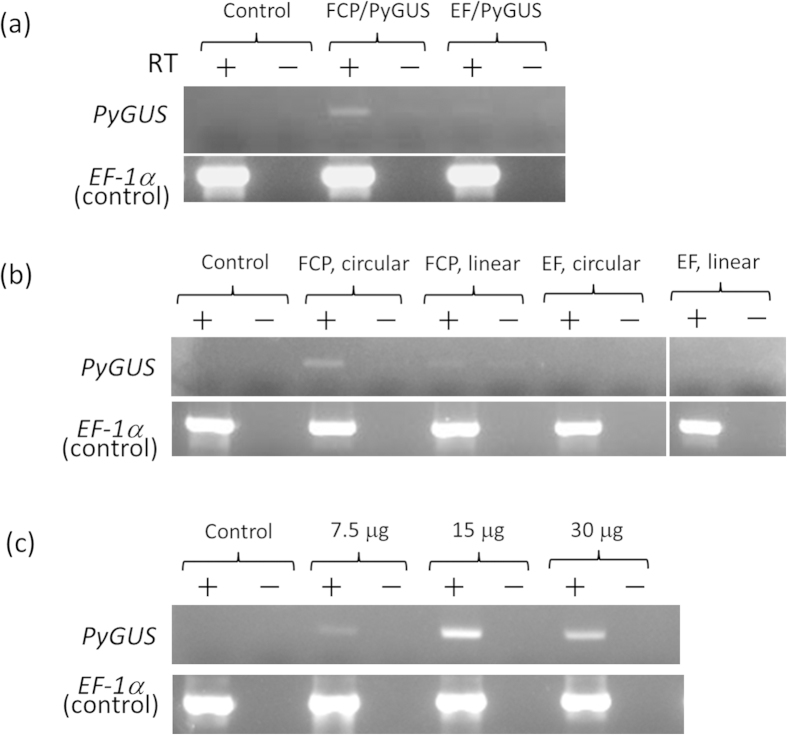
Analysis of transient PyGUS gene expression. **(a)** Comparison of *PyGUS* expression with the *FCP* promoter and *EF-1α* promoter. **(b)** Comparison of *PyGUS* expression depending on vector topology: the super-coiled (circular) form and linearized form (linear). **(c)** Relationship between the amount of vector used and *PyGUS* expression.

According to our preliminary results from expressed sequence tag (EST) and expression analyses of *P. carterae* (unpublished data), we selected the promoters of the fucoxanthin chlorophyll a/c-binding protein (*FCP*) and elongation factor-1α (*EF1α*) genes as candidates to drive the marker genes in a gene transformation system. The EST analysis also revealed that codon preference of *P. carterae* is highly GC-biased and closely resembles that of a macroalga, *Pyropia yezoensis* (Bangiales, Rhodophyta). Thus, *P. yezoensis* β-glucuronidase (*PyGUS*)^10^ and aminoglycoside phosphotransferase (*PyAph7*)^11^ were used as selectable marker genes without further modifications (Fig. 2). Three days after vector administration, the mRNA of *PyGUS* was clearly detected in the cells transferred with the *FCP* promoter-containing vector (pFGS), whereas the cells induced with the vector containing the *EF-1α* promoter (pEGS) showed very weak *PyGUS* expression (Figs 2a,b and 3a). This result prompted us to focus on the *FCP* promoter for the subsequent analyses. The topology of the introduced vector also affected the expression of marker genes. The supercoiled form (covalently closed and circular) of the vector resulted in relatively high expression compared to the linearized form (Fig. 3b). In general, the super-coiled plasmid form is desired for transfection since this can ensure efficient access to the nucleus of eukaryotic cells. For example, in the case of the diatom *Phaeodactylum tricornutum*, a substantially higher transformation frequency (approximately 30-fold greater) was confirmed with supercoiled DNA compared to linearized DNA^1^. The amount of the transferred plasmid DNA also affected the expression efficiency. In the present study, 15 and 30 μg of the plasmid DNA per 4.2 × 10^5^ cells showed the best result (Fig. 3c). Consistent with the results of the reverse transcription-polymerase chain reaction (RT-PCR) analysis described above, GUS staining revealed that the infected cells clearly expressed GUS enzyme. Approximately 10–20 positive cells (~0.0024–0.0048%) were detected in one experimental group (4.2 × 10^5^ cells) when 15 μg of the vector was introduced in the cells (Fig. 1c). Green fluorescent protein (GFP) expression was also successfully observed when another transient expression construct (pFGF) was introduced to the cells (Figs 1d and 2c). Furthermore, dose dependency was observed when the amount of the expression vector increased from 7.5 to 30 μg (Fig. 1e).

**Figure 4:**
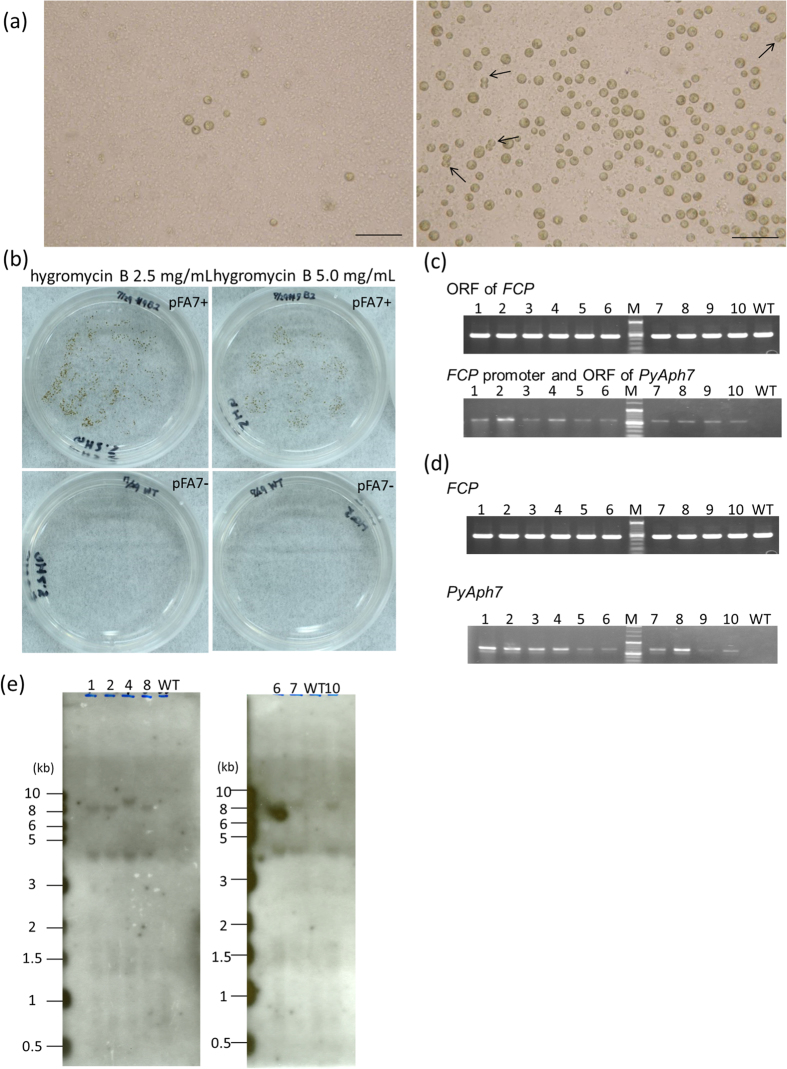
Antibiotic selection and gene expression of mutant strains. **(a)** Intact cells (left) and pFA7-introduced cells (right) after 3 weeks of selection on hygromycin B (2.5 mg/mL)-containing SLEP (representative image of n = 4). Arrows indicate the cells undergoing mitosis. Scale bars, 50 μm. **(b)** pFA7-introduced cells (upper panels) and intact cells (lower panels) on the hygromycin B-containing selective plates after 4 weeks of selection. Brown colonies were observed only in the transformed groups (representative image of n = 4). **(c)** Genomic PCR for ORF of endogenous *FCP* (upper), and for a region including the 3′-end of the *FCP* promoter and the 5′-end of the *PyAph7* ORF derived from pFA7 (lower) in the ten transformed mutant strains (1–10) and wild-type strain (WT); M, molecular marker. **(d)** Expression of *FCP* (upper) and *PyAph7* (lower) in the ten transformed mutant strains (1–10) and wild-type (WT) strain; M, molecular marker. **(e)** Southern hybridization for seven mutant strains and a WT strain; molecular marker bands are on the left.

Next, we tried to establish an antibiotic-resistant mutant strain using the pFA7 vector, which contains a codon-optimized hygromycin phosphotransferase gene (*PyAph7*) (Fig. 2d). Based on the results of the transient expression analysis described above, supercoiled DNA was used in the following experiments. Previously, Uji and colleagues succeeded in the stable nuclear transformation of *P. yezoensis* using *PyAph7* and hygromycin B. According to their report, we chose hygromycin B as a suitable selectable marker. In the hygromycin B-resistant test, the minimum inhibitory concentration (MIC) of *P. carterae* was estimated to be 1.0–1.5 mg/mL (Supplementary Fig. 1); the concentrations of the selective media were set to be slightly higher than the MIC to avoid false-positive results. The cells were tested at two concentrations of antibiotics, 2.5 and 5.0 mg/mL, in SLEP medium (see Methods). In both conditions, the intact cells (control experiment) were killed within 4–5 weeks, whereas the pFA7-introduced cells survived for more than 12 weeks. In addition, some cells were observed to undergo mitosis during the antibiotic selection experiment (Fig. 4a). The surviving cells were then transferred onto solid selective plates containing 2.5 or 5.0 mg/mL hygromycin B. As shown in Fig. 4b, the transformed cells survived on the plate and formed visible resistant colonies within 4 weeks. The cells that survived after selection with 2.5 mg/mL hygromycin B were considered to be antibiotic-resistant, and were used in subsequent experiments.

**Figure 5:**
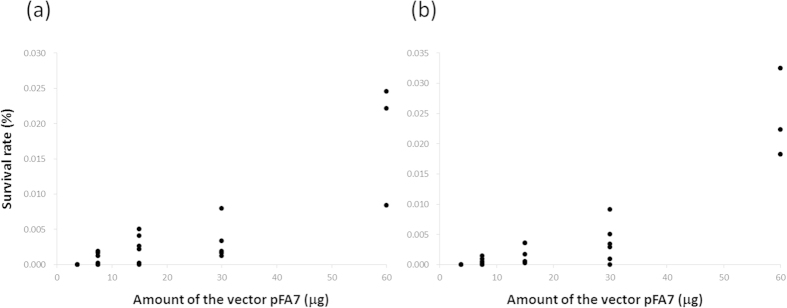
Survival rate analysis. The left and right panels represent colony numbers at 2 days **(a)** and 3 days **(b)** of preincubation before hygromycin B selection, respectively.

Ten surviving colonies were randomly selected from the selection plate to establish 10 mutant strains, inoculated in the conditioned SWEP medium (see Methods), and grown at a larger scale in non-selective SWEP medium for 8 weeks. To confirm that the transformed cell lines harbored the exogenous *PyAph7* gene, genomic PCR was performed with gene-specific primers. In all 10 strains, as well as the wild-type strain, the fragment of the open reading frame region derived from the endogenous *FCP* gene was detected at the expected size. However, the region including the *FCP* promoter and *PyAph7* derived from pFA7 was amplified only from the 10 transformed strains and not from the wild-type (Fig. 4c). This result indicates that the mutant strains maintained the exogenous *PyAph7* gene. RT-PCR analysis revealed that all of the mutant strains also expressed *PyAph7* under the non-selecting condition, whereas endogenous *FCP* expression was detected in all of the strains, including the wild-type (Fig. 4d). To verify the stable integration of *PyAph7* into the genome of the antibiotic-resistant strains, we conducted a Southern blot analysis using the seven fastest-growing mutants out of the ten strains described above. Hybridization with a *PyAph7*-derived probe revealed one or two hybridizing bands in all of the mutant strains (Fig. 4e). The larger signals were approximately 8 kb (strain Nos. 1, 2, 8, and 10), 9 kb (strain No. 4), and 7 kb (strain No. 6) in size. In addition, a band of approximately 4.3 kb was commonly observed in all of the mutant strains. Although the total length of the pFA7 construct (5.5 kb) is slightly larger than the position of the observed band, appearance of the band at 4.3 kb raised the possibility that the resistant construct might have remained as a circular plasmid in the mutant cells. Therefore, we conducted PCR analysis to amplify the flanking region of the *PyAph7* gene cassette using a primer set designed based on the pBI221 plasmid sequence. However, we could not detect any of the expected bands in the mutant strains, indicating that the strains did not carry the intact plasmids (Supplementary Fig. 2). These results indicated that at least 4 different mutant cell lines with distinct genotypes were obtained in this study. No hybridizing signals were observed in the wild type. Based on this result, the transformation efficiency is calculated to be 9.5 cells/10^6^ cells (4 mutant cell lines/4.2 × 10^5^ cells). To evaluate whether the transformation rate could be further increased by adding more plasmid DNA, the cells infected with the pFA7 vector were incubated in a non-selective medium for two or three days and then directly subjected to solid-plate selection. The number of surviving colonies increased remarkably in a dose-dependent manner within the range of the amount of the vector (Fig. 5).

## Discussion

Various methods for effective gene transfer in many algal species have been reported, including electroporation, biolistic DNA delivery (particle gun), and agitation with glass beads^8^,^12^. Among these techniques, the particle gun approach has recently been accepted as the most reliable, as it directly delivers DNA into the cells. In general, use of a gene gun results in higher transformation efficiency than PEG; however, this was not the case in our study. We detected transient *GUS* gene expression in the cells bombarded with pFGS vector, but failed to isolate hygromycin B-resistant strains using the pFA7 vector (data not shown). In the case of a gene gun, the expression vector is coated on the gold particle prior to bombardment. Thus, it is possible that the supercoiled plasmid DNA can be converted to a relaxed form during this step, resulting in low or unstable transformation efficiency. PEG-mediated transient or stable transformation is not as common as the other methods described above. However, effective gene transfer mediated by PEG has been reported in the green microalgae *Chlamydomonas reinhardtii*^13^ and *Chlorella ellipsoidea*^14^, and both of these studies were conducted with protoplasts or cell wall-deficient cells in logarithmic phase. We presume that the main obstacles that have hindered development of transgenic techniques for haptophytes thus far are their rigid calcified coccospheres and difficulties in controlling the proliferation rate. In this study, we have overcome these problems while developing a novel preparation method of protoplasts, which could easily enter the log phase of growth immediately after transfer to an appropriate medium.

The method presented here unambiguously meets the criteria for successful stable transformation, including successful integration of a foreign gene into the genome and expression of mRNA and functional translated products. Our next aim is to establish a more robust gene-manipulation tool such as a genome-editing technique. We believe that our newly developed method will provide valuable insight for achieving this next goal.

## Methods

### Cultivation of the coccolithophorid alga

*Pleurochrysis carterae* was grown in seawater-based Eppley’s medium^15^ (SWEP) for usual cultivation or in artificial seawater-based Eppley’s medium (SLEP) for the transformation experiments (Table 1). The culture conditions were 20 °C, with a photoperiod of 16-h light:8-h dark and no agitation or aeration. In usual cultivation, the medium was renewed every 4 weeks.

### Expression constructs

The sequences of the primers and the PCR conditions are summarized in Table 2. The upstream regions of *FCP* and *EF1α* were obtained by inverse PCR as follows. Genomic DNA was extracted from the wild-type strain of *P. carterae* using the Wizard Genomic DNA Purification Kit (Promega). The DNA was digested with *Pst*I for *FCP* or with *Xba*I for *EF1α*, and ligated using DNA Ligation Kit (TaKaRa). The first PCRs were carried out using the primer pairs FCPinvF4 and FCPinvR1 or EF1alINVF1 and EF1alINVR1. The nested PCR was carried out with the primer pairs FCPinvF5 and FCPinvR2 or EF1alINVF2 and EF1alINVR2. LA-Taq (TaKaRa) and KOD plus Neo (Toyobo) polymerases were used for *FCP* and *EF1α*, respectively. The PCR products of approximately 1,300 bp (*FCP*) and 2,700 bp (*EF1α*) were subcloned into the pGEM-T easy vector (Promega), and the sequences were determined. To prepare the expression constructs for transient expression, we used pBI221 as the basic vector. For transient expression of the *GUS* gene, the promoter region of pBI221 was replaced by the upstream region of *FCP* or *EF1α*(DDBJ accession nos. LC075595 and LC075596). Using the obtained sequences as the templates, approximately 1,200 bp of the promoter regions were amplified by PCR using FPCproLF1*Nco*I and FCPproLR2*Xba*I or EF1aproF1*Nco*I and EF1aproR1*Bam*HI, and subcloned into the corresponding sites of the vector. Then, the *GUS* gene was replaced by an artificial *PyGUS* gene, in which codon usage was adapted to that of the macroalga *Pyropia yezoensis* (Bangiales, Rhodophyta). For transient expression of the *GFP* gene, the vector was prepared by replacing the *GUS* gene with the *sGFP* gene amplified from pPyAct1-sGFP^16^ using the primers PcGFP1F1*Xba*I and PcGFP1R1*Sac*I. For stable expression, the expression construct was prepared using pEA7^11^ as the basic vector. The promoter region of *FCP* was obtained by PCR using FPCproLF1*Hind*III and PCPproLR2*Xba*I and subcloned into the *Hind*III-*Xba*I site of the pEA7 vector. These four expression constructs were designated as pFGS, pEGS, pFGF, and pFA7, respectively (Fig. 2). The vectors were propagated in the *Escherichia coli* strains XL-1blue or DH5*α*, and extracted using NucleoBond (Macherey-Nagel) immediately before analysis.

### PEG-mediated transfer

The cells were harvested from 50 mL of a 4–5-week-old culture by centrifugation at 110 × *g* for 5 min, and treated with proteinase K in SLEP (250 μg/mL) at 30 °C for 2 h and then at 20 °C for 2.0–2.5 h. To promote “molting” of the cell wall (coccosphere), the cells were treated with a hypo-osmotic buffer (Table 1)^9^ for 5–10 min in a 100-mm-diameter glass Petri dish, and removal of the coccosphere was carefully checked under the microscope. The protoplasts were then filtrated with three ply of Miracloth (EMD Millipore), collected by centrifugation at 60 × *g* for 3 min, and incubated in a 0.4 M mannitol solution at 4 °C for 20 min. After filtration through a tetron filter (180-mesh), approximately 4.2 × 10^5^ cells were resuspended in 320 μL of MaMg buffer (Table 1) and mixed with 7.5–60 μg of the expression vector or 15 μg of 500-kDa FITC-labeled dextran (SIGMA) diluted in 30 μL of double-distilled water. The same volume (350 μL) of a 40% PEG solution (Table 1) was added to the cell suspension and mixed by gentle shaking. PEG with a molecular weight of 6,000 (WAKO) was used throughout the present study. After 15 min of PEG reaction, the cells were washed with 5 mL of SLEP, collected by centrifugation at 60 × *g* for 5 min, and incubated in SLEP at 20  °C for 2 to 3 days.

### Transient expression of GUS and GFP

After 3 days of incubation, transient GUS expression was visualized with a 50 mM phosphate buffer (pH 7.0)-based GUS staining solution, containing 0.5 mM of X-Gluc, 0.5 mM of K_3_Fe(CN)_6_ and K_4_Fe(CN)_6_, 0.005% of TritonX-100, and 0.4 M mannitol. The cells were incubated at 25 °C for 16 h and stained cells were observed under the microscope. Expression of GFP was observed under a fluorescent microscope at 24 h after the PEG treatment.

### Isolation of antibiotic-resistant cells

To select for antibiotic-resistant cells, the cells were first cultured in SLEP containing 2.5 or 5.0 mg/mL of hygromycin B for 4 weeks. As a second selection step, the surviving cells were spread on a 0.2% gellan gum/45% SLEP plate containing 2.5 or 5.0 mg/mL of hygromycin B, and incubated until the colonies became visible and could be isolated (approximately 4–5 weeks). The colonies were picked and cultured in modified SLEP (SLEP with a 5-times concentration of vitamin mixture, 2.5 ppm of glycolic acid, and 0.625 mg/mL of ampicillin) for 3 days in a 96-well plate, and then transferred to a larger volume of SLEP in Petri dishes or glass flasks.

For the survival rate analysis, the cells infected with pFA7, at various amounts ranging from 0.375 to 60 μg, were directly restreaked onto the selective medium plate with 2.5 mg/mL of hygromycin B after 2 or 3 days of incubation. The number of colonies was counted after 3 weeks of selection.

### RT-PCR and genomic PCR

The sequences of the primers and the conditions used for PCR are summarized in Table 2. Transient expression of *PyGUS* was examined by RT-PCR. After 3 days of incubation, total RNA was extracted from the cells using RNeasy Plant Mini Kit (Qiagen), and treated with TurboDNase Kit (Ambion) to avoid contamination of genomic DNA. Using 50 ng of total RNA as a template, cDNA was synthesized with the poly-T_17_ primer and SuperScriptIII reverse transcriptase (Invitrogen). To quantify the expression level of *PyGUS and EF1α*, PCR was performed using LA-Taq polymerase with the primer pairs PyGUSExAF2 and PyGUSExAR2, and EF1aExF1 and EF1aExAR2. To examine the expression of *PyAph7*, total RNAs were extracted from the ten mutant strains, and cDNAs were prepared from 500 ng of total RNA as described above. The *Aph7* mRNA was amplified by PCR with the primers AphExAF1 and AphExAR2 using LA-Taq polymerase with GC buffer 2 (TaKaRa). Expression of endogenous *FCP* was also examined as a positive control using the primer pair FCPExAF2 and FCPExAR2.

Genomic DNA was extracted from the wild-type strain or hygromycin B-resistant strains with Chelex 100 resin (BioRad). The primers, checkFCPproF1232 and AphExAR1, for genomic DNA were designed to amplify the *FCP* promoter and *Aph7* gene, respectively (Fig. 2). As a control experiment, the open reading frame (ORF) of *FCP* was also amplified with the primers FCPExAF2 and FCPExAR2. Using 5 ng of genomic DNA as a template, PCR was carried out with LA-Taq polymerase and GC buffer 2.

### Southern blot analysis

Genomic DNAs were extracted from the eight fastest-growing strains, strains Nos. 1, 2, 4–8, and 10. The DNA samples (2.5 μg) were digested with *Bss*HII and transferred to a nylon membrane. The ^32^P-labeled DNA probe of *PyAph7* (G^3^ to T^692^, Fig. 2d) was prepared by a random-prime method and hybridized with the membrane. Signals were visualized using autoradiography.

## Additional Information

**How to cite this article**: Endo, H. *et al.* Stable Nuclear Transformation System for the Coccolithophorid Alga *Pleurochrysis carterae. Sci. Rep.* **6**, 22252; doi: 10.1038/srep22252 (2016).

## Supplementary Material

Supplementary Information

## Figures and Tables

**Table 1:** Media and solutions.

Eppley’s medium		
1000x metal stock	FeCl_3_ · 6H_2_O	1000 mg
	CuSO_4_ · 5H_2_O	4 mg
	Na_2_MoO_4_ · 4H_2_O	130 mg
	ZnSO_4_ · 7H_2_O	250 mg
	CoCl_2_ · 6H_2_O	4 mg
	MnSO_4_ · H_2_O	620 mg
	Na_2_EDTA	6 g
	fill up to	1000 mL
1000x NKP stock	KNO_3_	5.05 g
	K_2_HPO_4_	0.87 g
	fill up to	100 mL
1000x vitamine	thiamine	200 mg
	biotin	1 mg
	cyanocobalamin	10 mg
	fill up to	1000 mL
		
hypo-osmotic buffer	HEPES	10 mM
	KCl	100 mM
	NaOH	350 μM
MaMg buffer	mannitol	400 mM
(pH was adjusted to 5.8 with KOH)	MgCl_2_	15 mM
	MES	0.1% (w/v)
40% PEG in CMS solution	PEG	4 g
	CMS solution	6 mL
CMS solution	mannnitol	400 mM
(pH was adjusted to 7.0 with KOH)	Ca(NO_3_)_2_	100 mM

**Table 2:** Sequences of the primers and PCR programs.

Gene	Reaction	Primer(sense, 5′→3′)	Primer(antisense 5′→3′)	PCR condition
*FCP*	1st inverse PCR	FCPinvF4	FCPinvR1	94 °C 30 s, 50 °C 30 s, 72 °C 4 min
		CAT GTT GAT GAA GCC GAC GAG	TGC GCC GAA CTG TTC CGA T	25 cycles
	Nested inverse PCR	FCPinvF5	FCPinvR2	94 °C 30 s, 50 °C 30 s, 72 °C 4 min
		CGT TTT CGC CGG CAA TCG A	CCG AGA TCA CGT GGC ACA AGA T	25 cycles
	Subcloning of the promoter region	FCPproLF1NcoI	FCPproLR2XbaI	98 °C 10 s, 57 °C 30 s, 68 °C 90 s
		CCA TGG CTG CAT GCA GTA TCA ACA GGC A	TCT AGA CTC GCG CAT GGC TTC ACG AGT GTG TGT G	30 cycles
		FCPproLF1HindIII	FCPproLR2XbaI	98 °C 10 s, 55 °C 30 s, 68 °C 90 s
		AAG CTT CTG CAT GCA GTA TCA ACA GGC A	see above	25 cycles
	Expression analysis	FCPExAF2	FCPExAR2	94 °C 30 s, 58 °C 30 s, 72 °C 30 s
		ATG GCT CTC TCC CTG TCC G	CGA CCG TTG TTC AGC TCG AT	30 cycles
*EF1α*	1st inverse PCR	EF1alNVF1	EF1alNVR1	94 °C 10 s, 59 °C 30 s, 68 °C 2.5 min
		CCT CGA CAA GCA GAA CAT GCC	ATG GCG GTG GTG AAG TTA CC	25 cycles
	Nested inverse PCR	EF1alNVF2	EF1alNVR2	94 °C 10 s, 59 °C 30 s, 68 °C 2.5 min
		TCG ACA TTC CGG GCG AGA TC	GCG CCG GAG ATC ATG TTC TTG	25 cycles
	Subcloning of the promoter region	EF1aproF1NcoI	EF1aproR1BamHI	98°C 10 s, 57°C 30 s, 68°C 1 min
		CCA TGG CTG GGG CTG TTG CTG AGA TAC	GGA TCC CTT CTC CAT CTC ACG CTC CGG	27 cycles
				94°C 30 s, 52°C 30 s, 72°C for 40 s
				40 cycles
	Expression analysis	EF1aExAF1	EF1aExAR2	94 °C 30 s, 52 °C 30 s, 72 °C for 40 s
		CGA CAA CCT CAA CAA GAA GTC GAC	ATT GGC GAG TAG CCG AGC TT	40 cycles
	Genomic PCR	EF1aExAF1	EF1aExAR2	94 °C 30 s, 60 °C 30 s, 72 °C 30 s
		see above	see above	25 cycles
*GFP*	Subcloning of the ORF region	PcGFP1F1XbaI	PcGFP1R1SacI	98 °C 10 s, 57 °C 30 s, 68 °C 1 min
		TCT AGA ATG GTG AGC AAG GGC GAG	GAG CTC TTA CTT GTA CAG CTC GTC CAT G	27 cycles
*PyGUS*	Expression analysis	PyGUSExAF2	PyGUSExAR2	94 °C 30 s, 52 °C 30 s, 72 °C for 40 s
		GCA GTT CCT GAT CAA CCA CA	AGA ACA TCA CGT TCA CGC AC	40 cycles
*PyAph7*	Expression analysis	AphExAF1	AphExAR2	94 °C 30 s, 58 °C 30 s, 72 °C 45 s
		GAC GCA GGA GTC CCT GCT	ACG AAG ATG TTG GTC CCG T	30 cycles
*FCP* promoter -*PyAph7*	Genomic PCR	checkFCPproF1232	AphExAR1	94 °C 30 s, 60 °C 30 s, 72 °C 30 s
		ACA CTG CAC CGT CCA GGT T	TCC GGG AAG ACC TCG GAG T	30 cycles
