# Supplementary material for: Stable Nuclear Transformation System for the Coccolithophorid Alga Pleurochrysis carterae
Source: Sci Rep. 2016 Mar 7;6:22252. doi: 10.1038/srep22252 (PMC4779993; doi:10.1038/srep22252)
Supplement: Supplementary Information [file srep22252-s1.pdf]

Supplementary Information

Supplement to submitted manuscript titled:

Stable Nuclear Transformation System for the Coccolithophorid Alga *Pleurochrysis carterae*

Hirotoshi Endo*[1, 2, 3], Megumi Yoshida[3], Toshiki Uji[4], Naotsune Saga[5], Koji Inoue[3] & Hiromichi Nagasawa[2, 6]

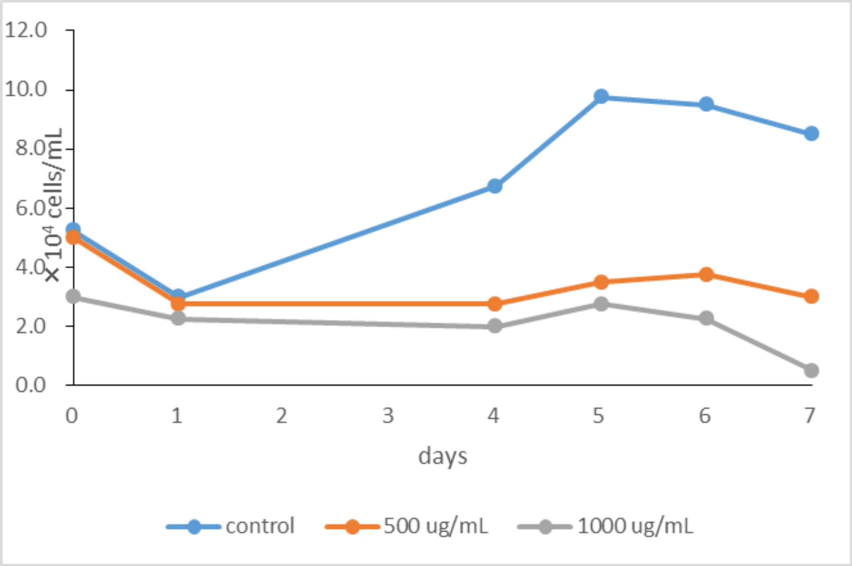

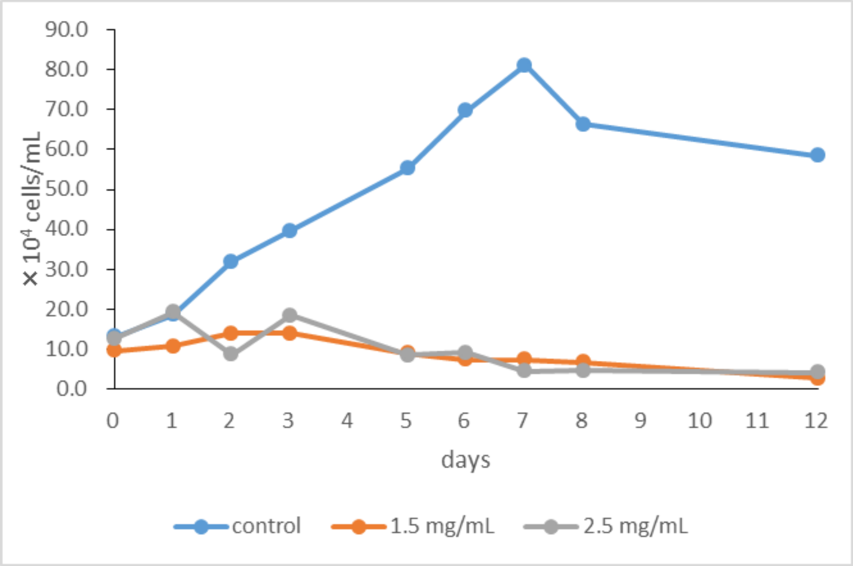

Supplementary Figure 1| Hygromycin B-resistance test of *P. carterae*.
Hygromycin B concentrations of 0.5, 1.0 mg/ml (a) and 1.5, 2.5 mg/ml (b) were tested in the liquid SLEP medium. The cells in the late logarithmic phase were used in the study.

Supplementary Figure 2

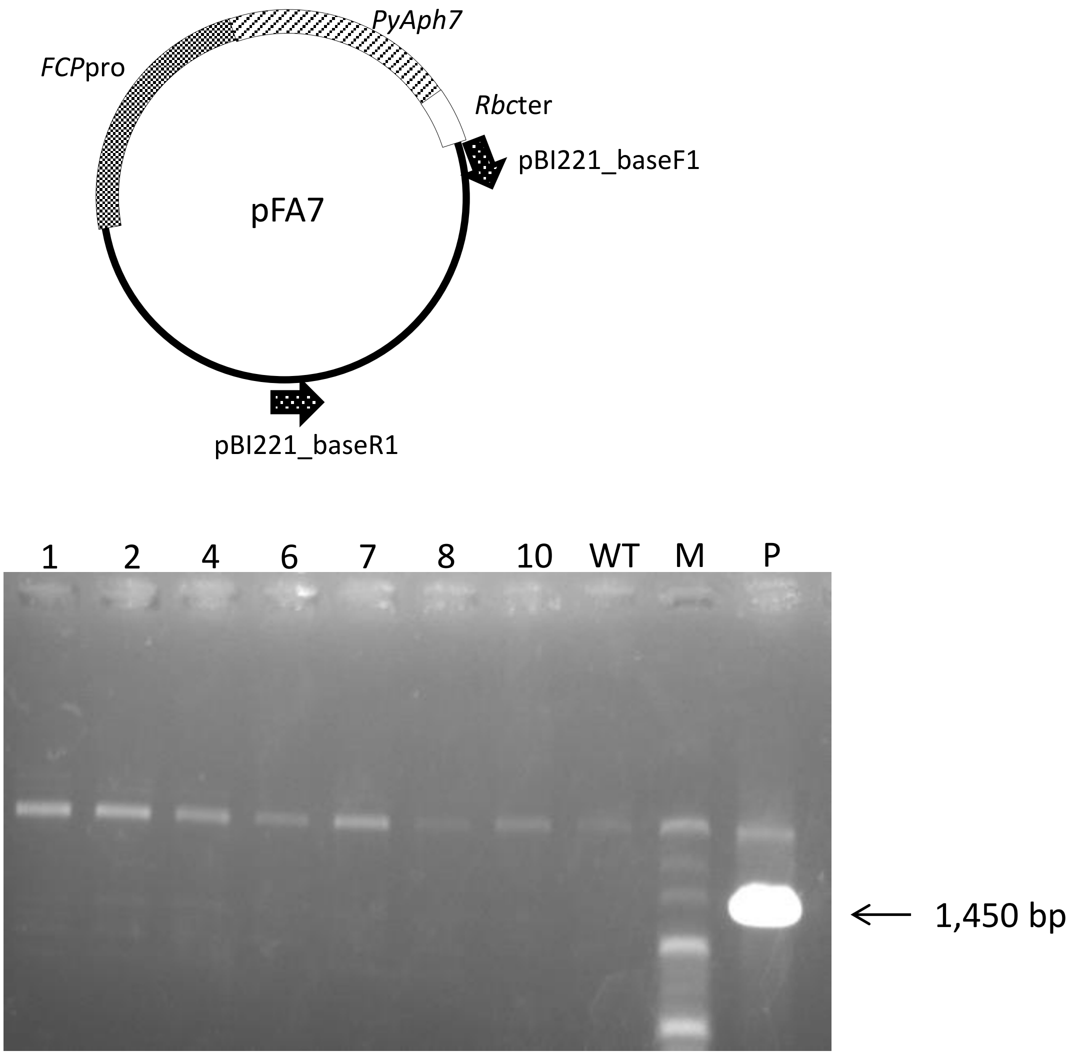

Supplementary Figure 2| Schematic of the expression construct pFA7 and agarose gel electrophoresis of PCR products amplifying the adjacent region to the CrRbcS terminator in pFA7 (Fig. 2).

The numerals represent the ID numbers of the mutant strains. WT, M, and P represent the wild type strain, molecular marker, and pFA7, respectively. PCR was carried out with the primers pBI221_baseF1 (5′-GAATTCACTGGCCGTCGTTT-3′) and pBI221_baseR1 (5′-AATCAGTGAGGCACCTATCTA-3′), under the following conditions:94°C 30 s, 54°C 30 s, 72°C 90 s, 35 cycles. A fragment of 1,450 bp derived from a circular vector template was amplified only in the positive control experiment (Lane P).
